# Supplementary material for: The Role of Capsule Endoscopy in the Diagnosis and Management of Small Bowel Tumors: A Narrative Review
Source: Cancers (Basel). 2024 Jan 7;16(2):262. doi: 10.3390/cancers16020262 (PMC10813471; doi:10.3390/cancers16020262)
Supplement: Supplementary file 1 [file cancers-16-00262-s001.zip › cancers-2744590-supplementary.pdf]

Table S1. Technical specifications of SB capsules from different manufacturers.

Company	Model	Dimensions (mm)	Weight (g)	Battery Life (h)	Image Sensor	Illumination	Field of View	Depth of Field (mm)	Image Sampling Rate (fps)	Adaptive Frame Rate	Real Time Monitoring
**Medtronic (Dublin, Ireland)**	Pillcam™ SB3	11 × 26	3.0	8	Front	4 LEDs	156°	0-30	2-6	Yes	Yes
**Olympus (Tokyo, Japan)**	Endo Capsule 10	11 × 26	3.3	12	Front	4 LEDs	160°	0-20	2	No	Yes
**Intromedic (Seoul, South Korea)**	Mirocam® MC 1600	10.8 × 24.5	3.25	12	Front	6 LEDs	170°	0-30	6	No	Yes
**Jinshan (Chongqing, China)**	OMOM® HD	11 × 25.4	3.0	12	Front	4 LEDs	172°	0-50	2 - 10	Yes	Yes
**Capsovision (Saratoga, CA, USA)**	CapsoCam Plus®	11 × 31	4	15	Lateral and circumferential	16 LEDs	360° lateral	0-18	Up to 5	Yes	No
